# Phylogenomics and signature proteins for the alpha Proteobacteria and its main groups

**DOI:** 10.1186/1471-2180-7-106

**Published:** 2007-11-28

**Authors:** Radhey S Gupta, Amy Mok

**Affiliations:** 1 Department of Biochemistry and Biomedical Science, McMaster University, Hamilton L8N3Z5, Canada

## Abstract

**Background:**

Alpha proteobacteria are one of the largest and most extensively studied groups within bacteria. However, for these bacteria as a whole and for all of its major subgroups (viz. *Rhizobiales, Rhodobacterales, Rhodospirillales, Rickettsiales, Sphingomonadales *and *Caulobacterales*), very few or no distinctive molecular or biochemical characteristics are known.

**Results:**

We have carried out comprehensive phylogenomic analyses by means of Blastp and PSI-Blast searches on the open reading frames in the genomes of several α-proteobacteria (viz. *Bradyrhizobium japonicum*, *Brucella suis*, *Caulobacter crescentus*, *Gluconobacter oxydans*, *Mesorhizobium loti*, *Nitrobacter winogradskyi*, *Novosphingobium aromaticivorans*, *Rhodobacter sphaeroides *2.4.1, *Silicibacter sp*. TM1040, *Rhodospirillum rubrum *and *Wolbachia *(*Drosophila*) endosymbiont). These studies have identified several proteins that are distinctive characteristics of all α-proteobacteria, as well as numerous proteins that are unique repertoires of all of its main orders (viz. *Rhizobiales, Rhodobacterales, Rhodospirillales, Rickettsiales, Sphingomonadales *and *Caulobacterales*) and many families (viz. *Rickettsiaceae, Anaplasmataceae, Rhodospirillaceae, Acetobacteraceae, Bradyrhiozobiaceae, Brucellaceae *and *Bartonellaceae*). Many other proteins that are present at different phylogenetic depths in α-proteobacteria provide important information regarding their evolution. The evolutionary relationships among α-proteobacteria as deduced from these studies are in excellent agreement with their branching pattern in the phylogenetic trees and character compatibility cliques based on concatenated sequences for many conserved proteins. These studies provide evidence that the major groups within α-proteobacteria have diverged in the following order: (*Rickettsiales*(*Rhodospirillales *(*Sphingomonadales *(*Rhodobacterales *(*Caulobacterales-Parvularculales *(*Rhizobiales*)))))). We also describe two conserved inserts in DNA Gyrase B and RNA polymerase beta subunit that are distinctive characteristics of the *Sphingomonadales *and *Rhodosprilllales *species, respectively. The results presented here also provide support for the grouping of *Hyphomonadacea*e and *Parvularcula *species with the *Caulobacterales *and the placement of *Stappia aggregata *with the *Rhizobiaceae *group.

**Conclusion:**

The α-proteobacteria-specific proteins and indels described here provide novel and powerful means for the taxonomic, biochemical and molecular biological studies on these bacteria. Their functional studies should prove helpful in identifying novel biochemical and physiological characteristics that are unique to these bacteria.

## Background

The α-proteobacteria form one of the largest groups within bacteria that includes numerous phototrophs, chemolithotrophs, chemoorganotrophs and aerobic photoheterotrophs [1]. They are abundant constituents of various terrestrial and marine environments [2]. *Pelagibacter oblique*, which is the smallest known free-living bacteria, is believed to be the most numerous bacteria on this planet (about 10^28 ^cells) comprising about 25% of all microbial cells in the oceans [2]. The intimate association that many α-proteobacteria exhibit with the eukaryotic organisms is of central importance from agricultural and medical perspectives [3,4]. Symbiotic association of the *Rhizobiaceae *family members with the plant root nodules is responsible for most of the atmospheric nitrogen fixation by plants [4-6]. Many other α-proteobacteria such as *Rickettsiales*, *Brucella *and *Bartonella *have adopted intracellular life styles and they constitute important human and animal pathogens [3,7-9]. Additionally, the α-proteobacteria have also played a seminal role in the origin of the eukaryotic cell [10,11].

In the current taxonomic scheme based on 16S rRNA, α-proteobacteria are recognized as a Class within the phylum Proteobacteria [1,12,13]. They are subdivided into 7 main subgroups or orders (viz. *Caulobacterales, Rhizobiales, Rhodobacterales, Rhodospirillales, Rickettsiales, Sphingomonadales *and *Parvularculales*) [12]. The α-proteobacteria and their main subgroups are presently distinguished from each other and from other bacteria primarily on the basis of their branching in phylogenetic trees [14,15]. However, we have previously described several conserved inserts and deletions (indels), as well as whole proteins, that are specific for these bacteria [16,17].

In the past 3–4 years, the numbers of α-proteobacterial genomes that have been sequenced has increased markedly. In addition to > 60 complete genomes (Table 1), sequence information is available for a large number of other species. These genomes cover all of the main groups within α-proteobacteria (Table 1) and provide an enormously valuable resource for identifying molecular characteristics that are unique to them. This information can be used to identify unique sets of genes or proteins that are distinctive characteristics of various higher taxonomic groups (e.g. families, orders, etc.) within α-proteobacteria. Such genes/proteins provide valuable tools for taxonomic, biochemical and molecular biological studies [17-26]. With this goal in mind, in the present work, we have performed comprehensive phylogenomic analyses of α-proteobacterial genomes to identify proteins/ORFs that are distinctive characteristics of the various higher taxonomic groups within α-proteobacteria. The phylogenomic distribution of these proteins is also compared with the branching patterns of these species in phylogenetic trees and in the character compatibility cliques to develop a reliable picture of α-proteobacterial evolution.

**Table 1:** Genome sizes, protein numbers and GC contents of sequenced alpha-proteobacteria

Species Name	Order	Family	Genome Size (Mb)	GC content (%)	Protein Number	Reference
*Bartonella bacilliformis *KC583	Rhizobiales	Bartonellaceae	1.4	38.2	1283	TIGR
*Bartonella henselae *str. Houston-1	Rhizobiales	Bartonellaceae	1.93	38.2	1488	[7]
*Bartonella quintana *str. Toulouse *	Rhizobiales	Bartonellaceae	1.58	38.8	1142	[7]
*Bradyrhizobium japonicum *USDA 110*	*Rhizobiales*	*Bradyrhizobiaceae*	9.1	64.1	8317	[5]
*Bradyrhizobium japonicum *BT Ail	*Rhizobiales*	*Bradyrhizobiaceae*	8.53	64.8	7622	DOE-JGI
*Bradyrhizobium sp*. ORS278	*Rhizobiales*	*Bradyrhizobiaceae*	7.5	65.5	6717	Genoscope
*Nitrobacter hamburgensis *X14	*Rhizobiales*	*Bradyrhizobiaceae*	5.01	61.6	4326	DOE-JGI
*Nitrobacter winogradskyi *Nb-255*	*Rhizobiales*	*Bradyrhizobiaceae*	3.4	2.5	3122	[42]
*Rhodopseudomonas palustris *BisA53	*Rhizobiales*	*Bradyrhizobiaceae*	5.51	64.4	4878	DOE-JGI
*Rhodopseudomonas palustris *BisB18	*Rhizobiales*	*Bradyrhizobiaceae*	5.51	65.0	4886	DOE-JGI
*Rhodopseudomonas palustris *BisB5	*Rhizobiales*	*Bradyrhizobiaceae*	4.89	64.8	4397	DOE-JGI
*Rhodopseudomonas palustris *CGA009	*Rhizobiales*	*Bradyrhizobiaceae*	5.47	65.0	4820	[41]
*Rhodopseudomonas palustris *HaA2	*Rhizobiales*	*Bradyrhizobiaceae*	5.33	66.0	4683	DOE-JGI
*Brucella abortus *biovar 1 str. 9–941	*Rhizobiales*	*Brucellaceae*	3.29	57.2	3085	[69]
*Brucella melitensis *16M	*Rhizobiales*	*Brucellaceae*	3.29	57.2	3198	[38]
*Brucella melitensis *biovar Abortus 2308	*Rhizobiales*	*Brucellaceae*	3.28	57.2	3034	[39]
*Brucella suis *1330*	*Rhizobiales*	*Brucellaceae*	3.32	57.3	2123	[40]
*Brucella ovis *ATCC 25840	*Rhizobiales*	*Brucellaceae*	3.3	57.2	2892	TIGR
*Mesorhizobium loti *MAFF303099*	*Rhizobiales*	*Phyllobacteriaceae*	7.6	62.5	7372	[6]
*Mesorhizobium *sp. BNC1	*Rhizobiales*	*Phyllobacteriaceae*	4.94	61.1	4543	DOE-JGI
*Agrobacterium tumefaciens *str. C58	Rhizobiales	Rhizobiaceae	5.67	59.0	4661	[37]
*Rhizobium etli *CFN 42	*Rhizobiales*	*Rhizobiaceae*	6.53	61.0	5963	[35]
*Rhizobium leguminosarum *bv. viciae 3841	*Rhizobiales*	*Rhizobiaceae*	7.75	61.0	7263	[81]
*Sinorhizobium meliloti 1021*	*Rhizobiales*	*Rhizobiaceae*	6.69	62.2	6205	[36]
*Caulobacter crescentus *CB15*	*Caulobacterales*	*Caulobacteraceae*	4.02	67.2	3737	[51]
*Hyphomonas neptunium *ATCC 15444	*Caulobacterales+*	*Hyphomonadaceae*	3.71	61.9	3505	[46]
*Maricaulis maris *MCS10	*Caulobacterales+*	*Hyphomonadaceae*	3.37	62.7	3063	DOE-JGI
*Jannaschia *sp. CCS1	*Rhodobacterales*	*Rhodobacteraceae*	4.4	62.2	4212	DOE-JGI
*Paracoccus denitrificans *PD1222	*Rhodobacterales*	*Rhodobacteraceae*	5.25	66.8	5077	DOE-JGI
*Rhodobacter sphaeroides *2.4.1*	*Rhodobacterales*	*Rhodobacteraceae*	4.45	68.8	4242	DOE-JGI
*Rhodobacter sphaeroides *ATCC 17025	*Rhodobacterales*	*Rhodobacteraceae*	4.54	68.2	4333	DOE-JGI
*Rhodobacter sphaeroides *ATCC 17029	*Rhodobacterales*	*Rhodobacteraceae*	4.42	69.0	4132	DOE-JGI
*Roseobacter denitrificans *OCh 114	*Rhodobacterales*	*Rhodobacteraceae*	4.3	58.9	4129	[44]
*Silicibacter pomeroyi *DSS-3	*Rhodobacterales*	*Rhodobacteraceae*	4.6	64.1	4252	[45]
*Silicibacter *sp. TM1040*	*Rhodobacterales*	*Rhodobacteraceae*	4.15	60.1	3864	DOE-JGI
*Gluconobacter oxydans *621H*	*Rhodospirillales*	*Acetobacteraceae*	2.92	60.8	2664	[56]
*Granulibacter bethesdensis *CGDNIH1	*Rhodospirillales*	*Acetobacteraceae*	2.7	59.1	2437	Rocky Mountain Lab
*Acidiphilium cryptum *JF-5	*Rhodospirillales*	*Acetobacteraceae*	3.97	67.1	3564	DOE-JGI
*Magnetospirillum magneticum *AMB-1	*Rhodospirillales*	*Rhodospirillaceae*	4.97	65.1	4559	[57]
*Rhodospirillum rubrum *ATCC 11170*	*Rhodospirillales*	*Rhodospirillaceae*	4.41	65.4	3841	DOE-JGI
*Candidatus Pelagibacter *ubique HTCC1062	*Rickettsiales*	*-*	1.3	29.7	1354	[2]
*Anaplasma marginale *str. St. Maries	Rickettsiales	Anaplasmataceae	1.2	49.8	949	[70]
*Anaplasma phagocytophilum *HZ	Rickettsiales	Anaplasmataceae	1.47	41.6	1264	[71]
*Ehrlichia canis *str. Jake	*Rickettsiales*	*Anaplasmataceae*	1.32	29.0	925	DOE-JGI
*Ehrlichia chaffeensis *str. Arkansas	*Rickettsiales*	*Anaplasmataceae*	1.18	30.1	1105	[71]
*Ehrlichia ruminantium *str. Gardel	*Rickettsiales*	*Anaplasmataceae*	1.5	27.5	950	[72]
*Ehrlichia ruminantium *str. Welgevonden	*Rickettsiales*	*Anaplasmataceae*	1.52	27.5	888	[73]
*Neorickettsia sennetsu *str. Miyayama	*Rickettsiales*	*Anaplasmataceae*	0.86	41.1	932	[71]
*Rickettsia bellii *RML369-C	*Rickettsiales*	*Rickettsiaceae*	1.52	31.6	1429	[74]
*Rickettsia conorii *str. Malish 7	*Rickettsiales*	*Rickettsiaceae*	1.27	32.4	1374	[75]
*Rickettsia felis *URRWXCal2	*Rickettsiales*	*Rickettsiaceae*	1.59	32.5	1512	[76]
*Rickettsia prowazekii *str. Madrid E	*Rickettsiales*	*Rickettsiaceae*	1.11	29.0	835	[77]
*Rickettsia typhi *str. Wilmington	*Rickettsiales*	*Rickettsiaceae*	1.11	28.9	838	[78]
*Wolbachia endosymbiont *(Drosophila)*	*Rickettsiales*	*Rickettsiaceae*	1.27	35.2	1195	[79]
*Wolbachia endosymbiont *(Brugia malayi)	Rickettsiales	*Rickettsiaceae*	1.08	34.2	805	[80]
*Erythrobacter litoralis *HTCC2594	*Sphingomonadales*	*Erythrobacteraceae*	3.05	63.1	3011	GBM Foundation
*Novosphingobium aromaticivorans *DSM 12444*	*Sphingomonadales*	*Sphingomona-daceae*	3.56	65.2	3937	DOE-JGI
*Sphingopyxis alaskensis *RB2256	Sphingomonadales	*Sphingomona-daceae*	3.37	65.5	3195	DOE-JGI
*Sphingomonas wittichii RW1*	Sphingomonadales	*Sphingomona-daceae*	5.93	67.9	5345	DOE-JGI
*Zymomonas mobilis *subsp. mobilis ZM4	*Sphingomonadales*	*Sphingomona-daceae*	2.06	46.3	1998	[53]

* indicates species that were used in blast searches

+ Although these species are classified as *Rhodobacterales*, results presented here and elsewhere [29,47] suggest their placement in the *Caulobacterales*.

DOE-JGI, Department of Energy, Joint Genome Institute; GBM, The Gordon and Betty Moore Foundation Marine Microbiology Initiative; TIGR, The Institute for Genome Research.

## Results and discussion

### Phylogeny of alpha proteobacteria

**Figure 1:**
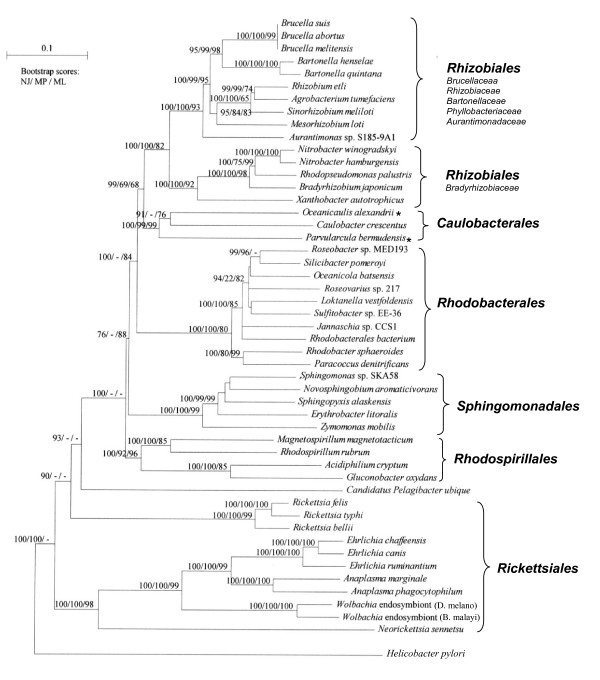
A neighbour-joining distance tree based on concatenated sequences for 12 conserved proteins. The numbers on the nodes indicate bootstrap scores (out of 100) observed in the neighbour-joining (NJ), maximum parsimony (MP) and maximum-likelihood (ML) analyses (NJ/MP/ML). The species marked with * are presently not part of the *Caulobacterales *order, but the results of phylogenetic and phylogenomic studies presented here suggest their placement in this group.

For comparing and interpreting the results of phylogenomic analysis, it was necessary at first to examine the evolutionary relationships among α-proteobacteria in phylogenetic trees. Phylogenetic analyses for α-proteobacteria was carried out based on concatenated sequences for 12 highly conserved proteins (see Methods). The relationships among these species were examined by both traditional phylogenetic methods (viz. neighbour-joining (NJ), maximum parsimony (MP) and maximum-likelihood (ML)) and by the character compatibility approach [27]. Figure 1 presents a neighbour-joining distance tree for α-proteobacteria, showing the bootstrap scores for various nodes using the NJ, MP and ML methods. In this tree, all of the main groups or orders within α-proteobacteria (viz. *Caulobacterales, Rhizobiales, Rhodobacterales, Rhodospirillales *and *Sphingomonadales*), except the *Rickettsiales*, formed well-resolved clades by different methods. For the *Parvularculales*, sequence information was available from a single species and it branched with the *Caulobacterales*. The two main families of the *Rickettsiales *i.e. *Rickettsiaceae *and *Anaplasmataceae *did not form a monophyletic clade, although they constituted the deepest branching lineages within α-proteobacteria. Except for the NJ method, the relative branching of different main groups within α-proteobacteria was not resolved by other methods. The branching orders of different groups as seen here is similar to that observed previously in the rRNA trees [1,15,16,28]. Recently, after this work was completed, Williams et al. [29] have reported similar results based on phylogenetic analysis of a different large set of protein sequences.

**Figure 2:**
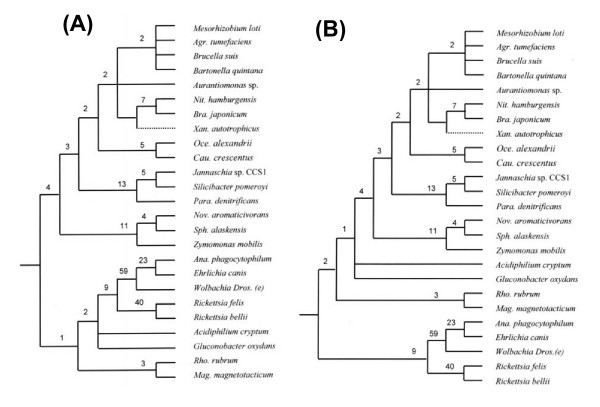
Character compatibility cliques showing the two largest cliques of mutually compatible characters based on the two states sites in the concatenated sequence alignment for 12 conserved proteins. The cliques consisted of 350 mutually compatible characters. The numbers of characters that distinguished different clades are indicated on the nodes. Rooting was done using the sequences for *Helicobacter pylori and Campylobacter jejuni*. *, as in Figure 1.

The evolutionary relationships among α-proteobacteria were also examined using the character compatibility approach [27]. This method removes all homoplasic and fast-evolving characters from the dataset [27,30,31] and it has proven useful in obtaining correct topology in cases which have proven difficult to resolve by other means [31-33]. These analyses were carried out on a smaller set of 27 species containing all main groups of α-proteobacteria and two ε-proteobacteria. The concatenated sequence alignment for the 12 proteins contained 896 positions that were useful for these studies (i.e. those sites where only two amino acids were found with each present in at least two species). The compatibility analysis of these sites resulted in 12 largest cliques each containing 350 mutually compatible characters. The two main relationships observed in these cliques are shown in Fig. 2. The other cliques differed from those shown only in the relative branching positions of various *Rhizobiaceae *species, which varied from each other by a single character and are shown as unresolved in Fig. 2. In both these cliques, the species from all main orders within α-proteobacteria were clearly distinguished by multiple unique characters. Further, in contrast to the phylogenetic trees where *Rickettsiaceae *and *Anaplasmataceae *did not form a distinct clade (Fig. 1), their monophyletic grouping was strongly supported by 9 unique shared characters (Fig. 2). The *Rickettsiales *and *Rhodospirillales *formed the deepest branching lineages in these cliques and other groups within α-proteobacteria branched in the following order: (*Sphingomonadales *(*Rhodobacterales *(*Caulobacterales *(*Rhizobiales*)))). This branching order was supported by multiple unique characters at each node giving confidence in the results.

The two main cliques obtained in these analyses differed from each other in terms of the branching position of the species belonging to the order *Rhodospirillales*. In one clique (Fig. 2A), the *Rhodospirillales *branched with the *Rickettsiales*, whereas in the other this group of species was found to branch after the *Rickettsiales *and it formed outgroup of the other α-proteobacteria (Fig. 2B). However, only a single character supported the former relationship indicating that it was not reliable. The two families within the *Rhodospirillales *order (*Rhodospirillaceae *and *Acetobacteraceae*), although they branched close to each other, no unique character common to them was identified, indicating that they are highly divergent. The exact branching position of the *Xanthobacter* was also not resolved in these cliques. In some cliques, it appeared as an outgroup of the *Bradyrhizobiaceae *(as shown by the dotted lines in Fig. 2), whereas in others it was placed in the middle of the *Rhizobiaceae *and *Bradyrhizobiaceae *families (as seen for the *Aurantiomonas*).

### Phylogenomic analyses of alpha proteobacteria

Table 1 lists some characteristics of various α-proteobacterial genomes that have been sequenced. The genomes vary in size from less than 1 Mb for *Neorickettsia sennetsu *to more than 9.0 Mb for *Bradyrhizobium japonicum*. To identify proteins that are distinguishing features of various higher taxonomic groups within α-proteobacteria, systematic Blastp searches were performed on each ORF in the genomes of *B. japonicum *USDA 110, *Brucella suis *1330, *Caulobacter crescentus *CB15, *Gluconobacter oxydans *621H, *Mesorhizobium loti *MAFF303099, *Nitrobacter winogradskyi *Nb-255, *Novosphingobium aromaticivorans *DSM 12444, *Rhodobacter sphaeroides *2.4.1, *Silicibacter sp*. TM1040, *Rhodospirillum rubrum *ATCC 11170 and *Wolbachia *(*Drosophila melanogaster*) endosymbiont (see Methods section). The genomes chosen covered all main taxonomic groups within the sequenced α-proteobacteria. These analyses have identified large numbers of proteins that are uniquely found in particular groups of α-proteobacteria. A brief description of these results is given below.

### Proteins that are distinguishing features of all (or most) alpha proteobacteria

**Figure 3:**
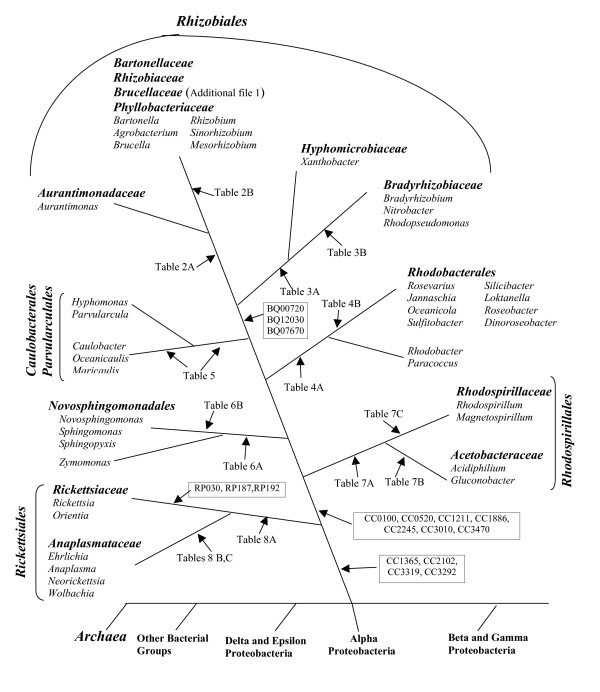
Summary of the phylogenomic analyses showing the species distribution of various α-proteobacteria-specific proteins and the suggested evolutionary stages where the genes for these proteins have likely evolved. The genes IDs for some proteins described in earlier work are indicated [17]. The information for all other proteins can be found in the indicated Tables or Additional files. A large numbers of conserved indels that are specific for different groups or clades within α-proteobacteria shown here have also been identified in our earlier work [16] (not shown here). The branching order of α-proteobacteria relative to other bacteria has been established in earlier work [32,58].

We previously described 6 proteins (viz. CC1365, CC1725, CC1887, CC2102, CC3292 and CC3319) that appeared distinctive characteristics of α-proteobacteria [17]. The α-proteobacterial specificity of these proteins in earlier work was only assessed by means of Blastp searches and it was not confirmed by PSI-Blast, as in the present work (see Methods). Further, since the earlier work, the number of sequenced α-proteobacteria and other genomes has more than doubled. Hence, it was important to confirm the α-proteobacteria specificity of these proteins. Our results reveal that four of these proteins viz. CC1365, CC2102, CC3292 and CC3319, are indeed specific for the α-proteobacteria as a whole, whereas for the remaining two proteins homologs showing significant similarities are also found in other bacteria. Of these four proteins, CC3292 is present in all sequenced α-proteobacteria including *Candidatus Pelagibacter ubique*, which is the smallest known free-living bacterium [2]. The protein CC2102 is only missing in *P. ubique*, while the other two proteins are only missing in 1–2 rickettsiae species (CC1365) and *P. ubique *(CC3319). These proteins, which are uniquely present in virtually all α-proteobacteria, provide distinguishing markers for this Class of bacteria (Fig. 3).

In earlier work, 9 proteins were identified that were present in nearly all α-proteobacteria, except the *Rickettsiales *[17]. These latter species are all intracellular bacteria that have lost many genes that are not required under these conditions [3,34]. The Blastp and PSI-Blast reexamination of these proteins have confirmed that 7 of these proteins (CC0100, CC0520, CC1211, CC1886, CC2245, CC3010 and CC3470) exhibit the indicated specificity. Except for CC0520 and CC3470, the other five proteins are also found in *P. ubique*, providing evidence for its placement within α-proteobacteria [2]. These results also provide evidence that *P. ubique *is not specifically related to the *Rickettsiales*. The phylogenomic distributions of these genes/proteins can be explained by either their evolution after the divergence of the *Rickettsiales *(Fig. 3), or by gene loss from this lineage.

### Proteins that are distinguishing features of the Rhizobiales species

The *Rhizobiales *species comprise more than 1/3^rd ^of the sequenced α-proteobacterial genomes (see Table 1). This order includes a wide assortment of species many of which interact with the eukaryotic organisms to produce diverse effects. This group includes various rhizobia (*Rhizobium, Mesorhizobium, Sinorhizobium*) and *Bradyrhizobia *species that induce root nodules in plants and live symbiotically within them to enable nitrogen fixation [4-6,35,36]. Another *Rhizobiaceae *species, *A. tumefaciens*, induces Crown gall disease (tumors) in plants [37]. *Bartonella *and *Brucella *species are intracellular pathogens responsible for a number of diseases in human and animals including trench fever and brucellosis [7,38-40]. Other members of this order exhibit enormous versatility in terms of their metabolic capabilities and life styles [1,41,42]. Earlier studies identified six proteins that appeared distinctive characteristics of the *Rhizobiales *species [17]. Reexamination of these proteins by the more stringent criteria used in the present work confirmed that three of these proteins (BQ00720, BQ07670 and BQ12030) are indeed specific for the *Rhizobiales *and they are present in virtually all sequenced species from this large order. In addition to the *Rhizobiales*, these proteins are also present in *Stappia aggregata*, which is presently grouped with the *Rhodobacterales *[12], but was originally known as a strain of *Agrobacterium *[43].

New blast searches on the genomes of *B. japonicum*, *B. suis*, *M. loti *and *N. winogradskyi *have identified large number of other proteins that are specific for different subgroups within the *Rhizobiales *order. Seven proteins listed in Table 2A are uniquely present in most of the sequenced species belonging to the *Rhizobiaceae *(*Rhizobium, Sinorhizobium, Agrobacterium*), *Brucellaceae, Phyllobacteriaceae (Mesorhizobium) *and *Aurantimonadaceae *families. The absence of many of these proteins in *Bartonella *species is probably due to gene loss [7,34]. These groups form a well-defined clade with high bootstrap scores in the NJ, MP and ML trees (see Fig. 1) and the genes for them likely evolved in a common ancestor of these genera/families (Fig. 3). Another 9 proteins (Table 2B) are uniquely present in most of the above species except *Aurantimonas*, which forms outgroup of the *Rhizobiaceae*, *Brucellaceae, Bartonellaceae *and *Phyllobacteriaceae *families (Figs. 1 and 2) [29]. Thus, the genes for these proteins have evolved after the branching of *Aurantimonadaceae *(Fig. 3). For six of the proteins in Tables 2A and 2B (marked with *), homologs with low E values are also found in *S. aggregata*, providing further evidence for its relatedness to the *Rhizobiaceae *family. Nine other proteins in Table 2C are found in most *Rhizobiaceae *species and *M. loti*, but they are missing in *Bartonella *and *Brucella *species. Although these proteins suggest a closer relationship between the *Rhizobiaceae *and *Phyllobacteriaceae *families, it is more likely that their genes have been lost from the *Bartonella *and *Brucella *species [34], which are intracellular bacteria. Additionally, some proteins were only found in either *Mesorhizobium *and *Rhizobium*, or *Mesorhizobium *and *Sinorhizobium *(Table 2D). These analyses have also identified 43 proteins that are uniquely present in all four sequenced *Brucella *species and many other proteins that are present in either three or two of the sequenced *Brucella *species (see Additional file 1).

**Table 2:** Proteins specific for the Rhizobiaceae and related species

Gene ID	Accession Number	Function	Gene ID	Accession Number	Function
**A. Proteins unique to *Aurantimonas, Mesorhizobium, Sinorhizobium, Rhizobium, Agrobacterium, Bartonella *and *Brucella***					

mll0062^1^	NP_101943	Hypothetical	mlr0789^1^	NP_102519	Hypothetical
mll4068^1^	NP_105027	Hypothetical	mlr3016	NP_104217	Omp10
mll7791^1,4^	NP_108034	Hypothetical	msl6526^1^	NP_107016	Hypothetical
mlr0777 ^1^	NP_102510	Hypothetical			

**B. Proteins unique to *Mesorhizobium, Sinorhizobium, Rhizobium, Agrobacterium, Bartonella *and *Brucella *(Missing in *Aurantimonas*)**					

mll0122 ^1,4^	NP_101988	Hypothetical	mll5001 ^1,4^	NP_105743	Hypothetical
mll1268 ^2^	NP_102895	Hypothetical	mll8359 ^1,4^	NP_108472	Hypothetical
mll2847 ^2,3^	NP_104087	Hypothetical	mlr1823 ^1^	NP_103319	Hypothetical
mll2898 ^4^	NP_104130	Hypothetical	mlr0094 ^1,4^	NP_101965	MhpC (COG0596)
mll4298 ^1,2^	NP_105201	Hypothetical			

**C. Proteins unique to *Mesorhizobium *and *Rhizobiaceae species***					

mll0080	NP_101954	Hypothetical	mll6703^4^	NP_107159	Hypothetical
mll0867	NP_102577	Hypothetical	mlr1904	NP_103376	Hypothetical
mll9619	NP_109472	Hypothetical	mlr3274	NP_104418	Hypothetical
mlr5174 ^4^	NP_105883	Hypothetical	mlr4951	NP_105704	NodF
mll6303	NP_106835	Hypothetical			

**D. Proteins specific to *Mesorhizobium *and either *Rhizobium *or *Sinorhizobium***					

mll0459	NP_102252	Hypothetical	mll2007	NP_103455	Hypothetical
mll1779	NP_103286	Hypothetical	mlr1999	NP_103450	Hypothetical
mll6195	NP_106741	Hypothetical	mlr2029	NP_103476	Hypothetical
mll8758	NP_106740	Hypothetical	mlr6601	NP_107075	Hypothetical
mlr3037	NP_104236	Transcriptional regulator			

^1 ^Missing in *Bartonella*

^2 ^Missing in *Agrobacterium*

^3 ^Missing in *Rhizobium*.

^4 ^Also found in *Stappia aggregata*

The analyses of proteins in the genomes of *B. japonicum *and *N. winogradskyi *have identified 12 proteins that are uniquely present in either all (or most) of the sequenced *Bradyrhizobiaceae *species as well as *X. autotrophicus *(Table 3A). The species from these two families form a strongly supported clade in the phylogenetic tree (Fig. 1)[29]. Sixty-two additional proteins in Table 3B are uniquely present in various species belonging to the *Bradyrhizobiaceae *family (i.e. *Bradyrhizobium, Nitrobacter, Rhodopseudomonas*). Many other proteins (see Additional file 2) are only found in two of the three *Bradyrhizobiaceae *genera and their distributions can result from gene losses, lateral gene transfers (LGTs), or other mechanisms.

**Table 3:** Proteins that are specific for the Bradyrhizobiaceae group

Gene ID	Accession Number	Function	Gene ID	Accession Number	Function
**A. Proteins Unique to *Bradyrhizobiaceae *Family and *Xanthobacter***					

bll6014^1,2^	NP_772654	Putative general secretion pathway protein M	Nwi_2179	YP_318785	Hypothetical
Nwi_1093	YP_317707	Hypothetical	Nwi_2432^1^	YP_319038	Hypothetical
Nwi_1227	YP_317841	Hypothetical	Nwi_2476^1^	YP_319081	Putative bacterioferritin
Nwi_1786^1^	YP_318399	Hypothetical	Nwi_2572	YP_319177	Hypothetical
Nwi_1788	YP_318401	Hypothetical	Nwi_2623	YP_319228	Hypothetical
Nwi_2147^1,3^	YP_318753	Hypothetical	Nwi_2707	YP_319312	Hypothetical

**B. Proteins Unique to *Bradyrhizobiaceae *Family**					

bll5899^2^	NP_772539	Hypothetical	Nwi_2021	YP_318632	Hypothetical
blr6106^1,2^	NP_772746	Hypothetical	Nwi_2063	YP_318673	Hypothetical
Nwi_0278	YP_316897	Hypothetical	Nwi_2064	YP_318674	Hypothetical
Nwi_0503	YP_317122	Hypothetical	Nwi_2163	YP_318769	Hypothetical
Nwi_0528	YP_317147	Hypothetical	Nwi_2173	YP_318779	Hypothetical
Nwi_0605^1^	YP_317224	Hypothetical	Nwi_2183^1^	YP_318789	Hypothetical
Nwi_0710^1,*d*^	YP_317328	Hypothetical	Nwi_2208^3^	YP_318814	Hypothetical
Nwi_0925	YP_317539	Hypothetical	Nwi_2244	YP_318850	Hypothetical
Nwi_0966^1,*d*^	YP_317580	Hypothetical	Nwi_2247	YP_318853	Hypothetical
Nwi_1084	YP_317698	Hypothetical	Nwi_2379	YP_318985	Hypothetical
Nwi_1092	YP_317706	Hypothetical	Nwi_2381^2^	YP_318987	Hypothetical
Nwi_1107^3^	YP_317721	Hypothetical	Nwi_2414^3^	YP_319020	Hypothetical
Nwi_1108	YP_317722	Hypothetical	Nwi_2489	YP_319094	Hypothetical
Nwi_1336	YP_317949	Hypothetical	Nwi_2492^13^	YP_319097	Hypothetical
Nwi_1139	YP_317753	Hypothetical	Nwi_2500	YP_319105	Hypothetical
Nwi_1247^3^	YP_317861	Hypothetical	Nwi_2506^3^	YP_319111	Hypothetical
Nwi_1270	YP_317883	Hypothetical	Nwi_2509^3^	YP_319114	Hypothetical
Nwi_1275^3^	YP_317888	Hypothetical	Nwi_2531	YP_319136	Hypothetical
Nwi_1454^1^	YP_318067	Hypothetical	Nwi_2575	YP_319180	Hypothetical
Nwi_1498	YP_318111	Hypothetical	Nwi_2577	YP_319182	Hypothetical
Nwi_1512	YP_318125	Hypothetical	Nwi_2588^13^	YP_319193	Hypothetical
Nwi_1581^3^	YP_318194	Hypothetical	Nwi_2630	YP_319235	Hypothetical
Nwi_1582	YP_318195	Hypothetical	Nwi_2676	YP_319281	Hypothetical
Nwi_1586	YP_318199	Dihydrofolate reductase	Nwi_2677^3^	YP_319282	Hypothetical
Nwi_1649^1,3^	YP_318262	Hypothetical	Nwi_2769	YP_319374	Hypothetical
Nwi_1674	YP_318287	Hypothetical	Nwi_2789^1^	YP_319394	Hypothetical
Nwi_1705	YP_318318	Hypothetical	Nwi_2984^3^	YP_319586	Hypothetical
Nwi_1711	YP_318324	Hypothetical	Nwi_2959	YP_319561	Hypothetical
Nwi_1785	YP_318398	Hypothetical	Nwi_3035	YP_319637	Hypothetical
Nwi_1793	YP_318406	Hypothetical	Nwi_3140	YP_319739	Hypothetical
Nwi_1800^1,2^	YP_318413	Hypothetical	Nwi_3141^1^	YP_319740	Hypothetical

^1 ^Missing in one or more strains of Rhodopseudomonas

^2 ^Missing in one or more species of Nitrobacter

^3 ^Missing in Bradyrhizobium japonicum USDA 110

### Proteins that are distinguishing features of the Rhodobacterales species

The order *Rhodobacterales *is a heterogeneous lineage of bacteria that exhibit much diversity in terms of their metabolism and cell division cycles [1,13]. This group includes many photosynthetic bacteria that are capable of CO_2 _as well as nitrogen fixation and also many chemoorganotrophs that can metabolize various sulfur-containing compounds [44,45]. A number of budding, stalk forming and prosthecate bacteria also belong to this group [46]. In addition to many completely sequenced genomes (see Table 1), information for several other species belonging to this order (e.g. *Sulfitibacter, Oceanicola, Loktanella, Jannaschia*, *Dinoroseobacter*, *Roseovarius *and *Sagittula*) is available in the NCBI database. To identify proteins that are specific for the *Rhodobacterales*, phylogenomic analyses were carried out on various ORFs in the genomes of *R. sphaeroides 2.4.1 *and *Silicibacter sp. TM1040*.

These studies have identified 29 proteins that are present in all-available *Rhodobacterales *species (Table 4A), but these proteins as well as those listed in Table 4B and 4C are not found in *H. neptunium, Oceanicaulis alexandrii*, *Maricaulis maris *or *Stappia aggregata*. These latter species are presently grouped with the *Rhodobacterales *[12], however, the absence of various *Rhodobacterales*-specific proteins in them and phylogenetic studies indicate that the placement of these species within this order is incorrect and needs be revised. In phylogenetic trees based on concatenated sequences for many proteins, *O. alexandrii *and *M. maris *consistently branched with the *Caulobacter *rather than the well-defined clade of *Rhodobacterales *(Figs. 1 and 2) [29]. The studies by Badger et al. [47] also provide strong evidence for the grouping of *H. neptunium *with the *Caulobacterales*.

**Table 4:** Proteins that are specific for the Rhodobacterales

Gene ID	Accession Number	Function	Gene ID	Accession Number	Function
**A. Proteins specific for *Rhodobacterales *(*Oceanicola, Loktanella, Paracoccus, Roseovarius, Roseobacter, Jannaschia, Silicibacte*r, *Sulfitobacter, Dinoroseobacter, Sagittula*)**					

TM1040_0093^1^	YP_612088	Hypothetical	TM1040_1842	YP_613837	Phasin, PhaP
TM1040_0184^2^	YP_612179	Hypothetical	TM1040_1967^3^	YP_613961	Putative CheA signal transduction
TM1040_0236^1,2^	YP_612231	Hypothetical	TM1040_1988	YP_613982	Hypothetical
TM1040_0471	YP_612466	Putative rod shape-determining protein MreD	TM1040_2263^2^	YP_614257	Hypothetical
TM1040_0586^2^	YP_612581	Hypothetical	TM1040_2370	YP_614364	Hypothetical
TM1040_0587^2^	YP_612582	Hypothetical	TM1040_2425^3^	YP_614419	Hypothetical
TM1040_0697	YP_612692	Hypothetical	TM1040_2466	YP_614460	GCN5-related N-acetyltransferase COG045
TM1040_0750^2,4^	YP_612745	Hypothetical	TM1040_2487^2^	YP_614481	Hypothetical
TM1040_0752^1^	YP_612747	Lipoprotein, putative	TM1040_2582^3^	YP_614576	Hypothetical
TM1040_1063	YP_613058	Gene transfer agent	TM1040_2999	YP_614993	Hypothetical
TM1040_1064	YP_613059	Gene transfer agent	TM1040_3077^3^	YP_611313	Hypothetical
TM1040_1247^3^	YP_613242	Hypothetical	TM1040_3749	YP_611978	Hypothetical
TM1040_1350^2^	YP_613345	Hypothetical	TM1040_3759	YP_611988	Lipoprotein, putative
TM1040_1406	YP_613401	Outer membrane chaperone Skp (OmpH)	TM1040_3764^3^	YP_611993	Putative transmembrane protein
TM1040_1567	YP_613562	Hypothetical			

**B. Proteins unique to various *Rhodobacterales *but missing in *Rhodobacter *and *Paracoccus***					

TM1040_1558^1^	YP_613553	Hypothetical	TM1040_2157^4^	YP_614151	Hypothetical
TM1040_1735^1^	YP_613730	Hypothetical	TM1040_2443^1,4^	YP_614437	Lipolytic enzyme, G-D-S-L
TM1040_1844^1,5,6^	YP_613839	Hypothetical	TM1040_2680^7^	YP_614674	Hypothetical

**C. Proteins Unique to *Silicibacter *and *Roseobacter***					

TM1040_1099^8^	YP_613094	Hypothetical	TM1040_3189	YP_611425	
TM1040_1423^8^	YP_613418	Hypothetical	TM1040_3202^8^	YP_611438	
TM1040_1451	YP_613446	Hypothetical	TM1040_3208^8^	YP_611444	
TM1040_1986^8^	YP_613980	Hypothetical	TM1040_3226^8^	YP_611462	
TM1040_2106	YP_614100	Hypothetical	TM1040_3529^8^	YP_611763	
TM1040_2139^8^	YP_614133	Hypothetical	TM1040_3626^8^	YP_611855	
TM1040_3075^8^	YP_611311	Hypothetical			

^1 ^Missing in Loktanella vestfoldensis SKA53

^2 ^Missing in one or more Rhodobacter sphaeroides strains

^3 ^Missing in Paracoccus denitrificans PD12222

^4 ^Missing in Rhodobacterales bacterium HTCC2654

^5 ^Missing in one or more species of Roseovarius

^6 ^Missing in Oceanicola batsensis HTCC2597

^7 ^Missing in one or more species of Roseobacter

^8 ^Missing in Silicibacter pomeroyi DSS-3

Six additional proteins in Table 4B are present in most of the *Rhodobacterales *species, but they are missing in *R. sphaeroides *and *P. denitrificans*, which form a distinct clade that appears as the outgroup of other *Rhodobacterales *species (Fig. 1)[29]. Thus, the genes for these proteins have likely evolved after the branching of these two genera. Thirteen additional proteins, which are only found in *Silicibacter *and *Roseobacter *genera (Table 4C) support a close relationship among them, as seen in phylogenetic trees (Fig. 1).

Of the proteins that are specific for *Rhodobacterales*, two of them (YP_613058 and YP_613059 in Table 4A, corresponding to proteins ABK27256 and ABK27255 in *R. capsulatus *genome) were previously identified as part of a complex referred to as gene transfer agent [48], based on similarity to certain virus-like elements. Another protein in the same category (viz. ABK27253) is specific for the *Rhodobacter *genus. The significance of these results is presently unclear.

### Proteins that are distinctive characteristics of the Caulobacterales

The order *Caulobacterales *is comprised of a single family with only four genera [12,49]. These chemoorganotrophic bacteria are commonly found in marine aerobic environments and they are distinguished by their ability to form stalked cells and unusual cell division cycle [49,50]. The complete genome of only *C. crescentus*, which is the best-studied organism from this group, is presently available [51]. However, as discussed above, a number of other species which are presently classified as *Rhodobacterales *viz. *O. alexandrii*, *M. maris*, *H. neptunium *and also *Parvularcula bermudensis*, consistently branch with *C. crescentus *in different phylogenetic trees (Figs. 1 and 2) [29,47]. Thus, phylogenomic analysis of the ORFs from *C. crescentus *genome was of much interest.

These analyses have identified 2 proteins (CC0486 and CC2480), which are uniquely present in this species as well as *O. alexandrii*, *M. maris*, *H. neptunium *and *P. bermudensis *(Table 5) One additional protein, CC2764 is present in all of these species except *H. neptunium*. The remaining eight proteins in Table 5 are only found in *C. crescentus*, *O. alexandrii *and *M. maris*, indicating that the latter two species are more closely related to *C. crescentus *in comparison to either *H. neptunium *or *P. bermudensis*. These results are strongly supported by the branching patterns of these species in phylogenetic trees (Figs. 1 and 2) [29,47]. Previously, Badger et al. [46] have reported identification of 62 proteins that were only found in *C. crescentus *and *H. neptunium*. However, the blast threshold used in this study to infer the absence of these proteins in other species was very high i.e. 1e-10. By the criteria used in the present work (see Methods), none of these 62 proteins was found to be unique to these two species.

**Table 5:** Proteins specific for the Caulobacter and related species

**Proteins Unique to *Caulobacter, Oceanicaulis *and *Maricaulis *(Some also found in *Hyphomonas *and *Parvularcula*)**					
Gene ID	Accession Number	Function	Gene ID	Accession Number	Function

CC0486^1^	NP_419305	Hypothetical	CC1066	NP_419882	Hypothetical
CC2480^1^	NP_421283	Hypothetical	CC1586	NP_420397	Hypothetical
CC2764^2^	NP_421560	Hypothetical	CC2207	NP_421010	Hypothetical
CC3101	NP_421895	Hypothetical	CC2628	NP_421428	hfaA protein
CC0512	NP_419331	Hypothetical	CC2639	NP_421438	Hypothetical
CC1064	NP_419880	Hypothetical			

All of the proteins in this Table are present in *C. crescentus *as well as in *O. alexandrii *and *M. maris*.

^1 ^These proteins are also present in *H. neptunium *and *P. bermudensis*.

^2 ^Also found in *P. bermudensis*.

### Proteins that are distinguishing characteristics of the Sphingomonadales

The species belonging to the order *Sphingomonadales *are present in both terrestrial and aquatic environments [52]. A distinguishing characteristic of many species from this group is the presence of glycosphingolipids in their cell envelope rather than lipopolysaccharides [52,53]. Several species from this group (e.g. *N. aromaticivorans*) can degrade a wide variety of aromatic hydrocarbons [52], whereas others such as *Zymomonas mobilis*, can highly efficiently ferment sugar to ethanol [53], making them of much interest and importance from biotechnological standpoints. This group also includes phototrophic organisms (e.g. *Erythrobacter litoralis*), which contain bacteriochlorophyll *a *and can derive significant fraction of their metabolic energy via anaerobic photosynthesis [54]. The complete genomes of 5 species from this order are now available (see Table 1). In addition, large numbers of sequences for *Sphingomonas sp. SKA58*, are also available in the NCBI database. Blast searches on the ORFs in the genome of *N. aromaticivorans *have identified 16 proteins (Table 6A) that are uniquely present in all 6 of the *Sphingomonadales *species for which information is available. Thirteen additional proteins (Table 6B) are present in all other *Sphingomonadales *species, except *Z. mobilis*, which is the deepest branching species within this group (Fig. 1). The genes for these proteins likely evolved after the branching of *Z. mobilis*. Many other proteins are present in only 3 or 4 of these species (viz. *N. aromaticivorans, E. litoralis*, *S. alaskensis, S. wittichiii *and *Sphingomonas sp. SKA58*) (see Additional file 3) and their phylogenomic distribution can result from a variety of mechanisms including shared ancestry, gene loss and LGTs among these species.

**Table 6:** Proteins that are specific for the *Sphingomonadales *group of species

Gene ID	Accession Number	Function	Gene ID	Accession Number	Function
**A. Proteins Unique to *Sphingomonadales *Order (Novosphingobium, Erythrobacter, Sphingomonas, Sphingopyxis and Zymomonas)**					

Saro_0018	YP_495301	Hypothetical	Saro_1291	YP_496569	Hypothetical
Saro_0052^1^	YP_495335	Hypothetical	Saro_1378	YP_496656	Hypothetical
Saro_0087	YP_495370	Hypothetical	Saro_1914^1^	YP_497188	Hypothetical
Saro_0150	YP_495433	Hypothetical	Saro_2130	YP_497403	Hypothetical
Saro_0232	YP_495514	Hypothetical	Saro_2788	YP_498058	Hypothetical
Saro_0409	YP_495691	Hypothetical	Saro_2958	YP_498227	Hypothetical
Saro_1088	YP_496367	Hypothetical	Saro_3138	YP_498407	Hypothetical
Saro_1144	YP_496423	Hypothetical	Saro_3213	YP_498482	Hypothetical

**B. Proteins Unique to *Sphingomonadales *but missing in *Zymononas***					

Saro_0044	YP_495327	Hypothetical	Saro_1748^2^	YP_497022	
Saro_0154	YP_495437	Hypothetical	Saro_1785	YP_497059	
Saro_0415^3^	YP_495697	Hypothetical	Saro_1972	YP_497246	
Saro_0458	YP_495740	Hypothetical	Saro_2036	YP_497309	
Saro_1078	YP_496357	Hypothetical	Saro_2037	YP_497310	
Saro_1126	YP_496405	Hypothetical	Saro_2333	YP_497604	
Saro_1160	YP_496439	Hypothetical	Saro_2548	YP_497818	
Saro_1163	YP_496442	Hypothetical			

^1 ^Missing in *Sphingomonas wittchii*

^2 ^Blast scores for *C. crescentus *and *H. neptunium *are also significant

^3 ^Significant blast scores for *C. crescentus*

**Figure 4:**
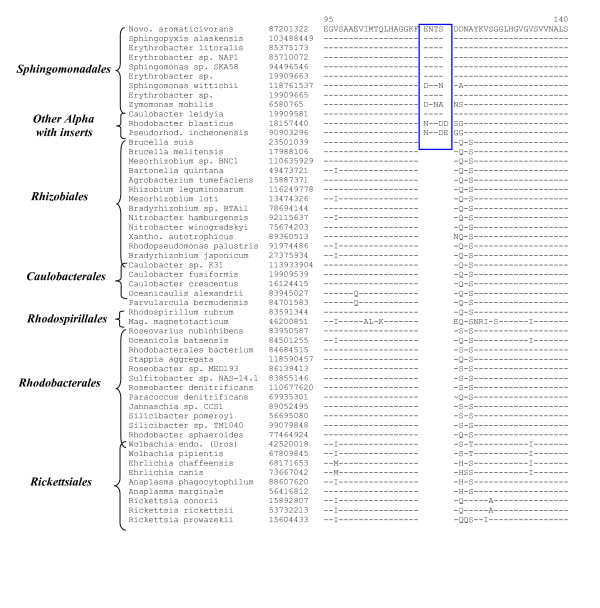
Partial sequence alignments of DNA Gyrase B showing a 4 aa insert that is mainly specific for the *Sphingomonadales *species. A 4–5 aa insert present in some other α-proteobacteria could be due to either LGTs or taxonomic anomalies. The dashes (-) denote identity with the amino acid on the top line. Sequence information for other groups of bacteria (which do not contain this insert) is not shown.

We have also identified a 4 aa insert in a highly conserved region of Gyrase B that is mainly specific for the species from this order (Fig. 4). This indel is present in all available *Sphingomonadales *species, but it is not found in most other alpha proteobacteria or other bacteria (results not shown). Besides *Sphingomonadales*, a similar size indel is also present in three other species (viz. *C. leidyia, R. blasticus*, and *Pesudorhodobacter incheonensis*). Because other *Rhodobacterales *or *Caulobacter *species lack this insert, the presence of this indel in these three species could be either due to LGTs or possibly due to taxonomic misclassification of these species.

### Proteins and indels that are specific for the Rhodospirillales species

**Figure 5:**
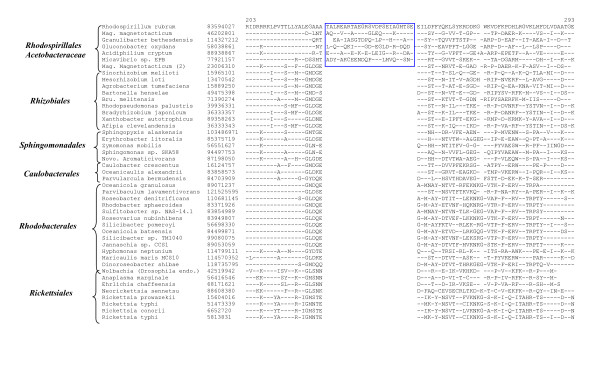
Partial sequence alignments of RNA polymerase β subunit (RpoB) showing a large insert (boxed) that is a distinctive characteristic of various *Rhodospirillales *species and not found in any other bacteria. There are two homologs of RpoB in *Magentospirillum (Mag.) magneticum *and only one of these contains the insert. The dashes (-) denote identity with the amino acid on the top line.

The order *Rhodospirillales *is comprised of diverse species including some photosynthetic and magnetotactic bacteria, some acidophiles as well as other bacteria commonly associated with flowers, fruits and fermented beverages that are involved in the partial oxidation of carbohydrates and alcohols [55]. The order is made up of two main families, *Rhodospirillaceae *and *Acetobacteraceae *[12,55]. The complete genomes of four species, two from each family, *Rhodospirillaceae *(*Magnetospirillum magnticum, R. rubrum*) and *Acetobacteraceae *(*Acidiphilium cryptum *and *G. oxydans*), are available (Table 1) [56,57]. Phylogenomic analyses of various ORFs in the genomes of *G. oxydans *and *R. rubrum *have led to identification of one proteins, GOX0963, which is uniquely found in all of these species (Table 7A). Three other proteins in this Table are present in at least 3 of the 4 species from this order. This table also lists 14 proteins each that are distinctive characteristics of either the *Acetobacteraceae *(Table 7B) or the *Rhodospirillaceae *(Table 7C) families, providing molecular markers for these families. We have also identified a 25 aa insert in a conserved region of the RNA polymerase beta subunit (RpoB) that is unique to various sequenced *Rhodospirillales *species, but not found in any other bacteria (Fig. 5).

**Table 7:** Proteins that are specific for the *Rhodospirillales *group

Gene ID	Accession Number	Function	Gene ID	Accession Number	Function
**A. Proteins Unique to *Rhodospirillales *Order (*Gluconobacter, Magnetospirillum, Rhodospirillum *and *Acidiphilium*)**					

GOX0633^1^	AAW60410	Hypothetical	GOX0963	AAW60735	Hypothetical
GOX0695^2^	AAW60472	Hypothetical	GOX1258^3^	AAW61019	Hypothetical

**B. Proteins Unique to *Acetobacteraceae *Family (*Gluconobacter *and *Acidiphilium*)**					

GOX0143	AAW59936	Hypothetical	GOX1616	AAW61357	Hypothetical
GOX0343	AAW60126	ANK, ankyrin repeats	GOX2216	AAW61951	Hypothetical
GOX1212	AAW60973	Phage portal protein	GOX2275	AAW62008	Hypothetical
GOX1215	AAW60976	Putative phage protein	GOX2316	AAW62049	Hypothetical
GOX1222	AAW60983	Putative phage protein	GOX2452	AAW62183	Hypothetical
GOX1224	AAW60985	Putative phage protein	GOX2454	AAW62185	Hypothetical
GOX1233	AAW60994	Hypothetical	GOX2456	AAW62187	Hypothetical

**C. Proteins Unique to *Rhodospirillaceae *Family (*Rhodospirillum *and *Magnetospirillum*)**					

Rru_A0125	YP_425217	Putative diguanylate phosphodiesterase	Rru_A2592	YP_427676	Hypothetical
Rru_A0152	YP_425244	Hypothetical	Rru_A2828	YP_427912	Hypothetical
Rru_A0531	YP_425622	Hypothetical	Rru_A3562	YP_428643	Hypothetical
Rru_A1689	YP_426776	Hypothetical	Rru_A3636	YP_428717	Hypothetical
Rru_A1756	YP_426843	Hypothetical	Rru_A3662	YP_428743	Hypothetical
Rru_A2112	YP_427199	Hypothetical	Rru_A3739	YP_428820	Hypothetical
Rru_A2510	YP_427597	Predicted transcriptional regulator	Rru_A3800	YP_428881	Hypothetical

^1 ^Missing in *Rhodospirillum*

^2 ^Missing in *Acidiphilium*

^3 ^Missing in *Magnetospirillum*

### Proteins that are specific for the Rickettsiales

The *Rickettsiales *species are intracellular pathogens responsible for a number of diseases in humans and other animals [3,34]. This order is comprised of three families, *Rickettsiaceae, Anaplasmataceae *and *Holosporaceae*. All of the sequenced genomes are from the first two families. Phylogenomic analysis of various ORFs from the genome of *Wolbachia *(*D. melanogaster*) endosymbiont has identified 3 proteins viz. WD0161, WD0715 and WD0771 (Table 8A) that are specific for the entire *Rickettsiales *order. Five other proteins in Table 8B (viz. WD0083, WD0157, WD0821, WD0827 and WD0863) are present in all sequenced *Ehrlichia, Anaplasma, Wolbachia *and *Neorickettsia *species (belonging to the *Anaplasmataceae *family), but they are not found in any of the *Rickettsiaceae *or other bacteria. Ten additional proteins (Table 8C) are uniquely present in the *Ehrlichia, Anaplasma *and *Wolbachia *species, but are absent in *Neorickettsia*. In view of the deep branching of *Neorickettsia *in comparison to these other genera (Fig. 1), the genes for these proteins have likely evolved after the branching of *Neorickettsia*. In earlier work, a number of proteins that appeared specific for the *Rickettsiaceae *family were also identified [17]. Additional Blastp and PSI-Blast searches on these proteins confirm that three of these proteins viz. RP030, RP187 are RP192, are indeed specific for the *Rickettsiaceae *family. Of these, RP030 is also found in *Orientia tsutsugamushi*.

**Table 8:** Protein that are specific for the Rickettsiales group of species

Gene ID	Accession Number	Function	Gene ID	Accession Number	Function
**A. Proteins Unique to *Rickettsiales *(*Wolbachia, Ehrlichia, Anaplasma, Rickettsia *and *Neorickettsia*)**					

WD0161	NP_965979	Hypothetical	WD0771^1^	NP_966526	Hypothetical
WD0715	NP_966474	Hypothetical			

**B. Proteins Unique to *Anaplasmataceae *Family (*Wolbachia, Ehrlichia, Anaplasma *and *Neorickettsia*)**					

WD0083	NP_965909	Hypothetical	WD0827	NP_966580	Hypothetical
WD0157	NP_965975	Hypothetical	WD0863	NP_966613	Hypothetical
WD0821	NP_966574	Hypothetical			

**C. Proteins Unique to *Anaplasmataceae *Family but missing in *Neorickettsia***					

WD0148	NP_965966	Hypothetical	WD0772	NP_966527	Hypothetical
WD0412	NP_966202	Hypothetical	WD1025	NP_966750	Hypothetical
WD0467	NP_966253	Preprotein translocase, SecG	WD1056	NP_966779	Hypothetical
WD0757	NP_966513	Hypothetical	WD1220	NP_966932	Hypothetical
WD0764	NP_966520	Hypothetical	WD1230	NP_966942	Hypothetical

^1 ^Missing in *Neorickettsia *and *Orientia*

## Conclusion

In this work, we have used a combined phylogenetic and phylogenomic approach to examine the evolutionary relationships among α-proteobacteria. Our analyses have identified large numbers of genes/proteins that are uniquely found in α-proteobacteria at various phylogenetic depths (Fig. 3). These include several proteins that are distinctive characteristics of all α-proteobacteria, as well as many proteins that constitute the unique repertoires of either all of the main orders of α-proteobacteria (viz. *Rhizobiales, Rhodobacterales, Rhodospirillales, Rickettsiales, Sphingomonadales *and *Caulobacterales*) or its different families (viz. *Rickettsiaceae, Anaplasmataceae, Rhodospirillaceae, Acetobacteraceae, Bradyrhizobiaceae, Brucellaceae *and *Bartonellaceae*). In addition, numerous other α-proteobacteria-specific proteins are present at different phylogenetic depths and they provide important information regarding the evolution of these bacteria. This work also describes two novel conserved indels in important housekeeping genes (viz. Gyrase B and RpoB) that are distinctive characteristics of the *Sphingomonadales *and *Rhodospirillales *orders, respectively. These indels are in addition to many other α-proteobacteria-specific indels that have been described in earlier work [16,58].

Based upon these α-proteobacteria-specific proteins and conserved indels, it is now possible to define nearly all of the higher taxonomic groups (i.e. most orders and many families) within α-proteobacteria in clear and definitive molecular terms based upon multiple characteristics (Fig. 3). The species distribution profiles of these α-proteobacteria-specific proteins and indels also provide important information regarding their branching order and interrelationships, which are highly concordant with each other (c.f. Fig. 26 in ref. [16] with Fig. 3 in this work). Importantly, the relationships that emerge from these phylogenomic analyses (Fig. 3) are in excellent agreement with the branching patterns of these species in different phylogenetic trees (Figs. 1 and 2) [29], giving high degree of confidence in the derived inferences. It should be noted that both in our work (Fig. 1) and that by Williams et al. [29], when analyses were performed using the traditional phylogenetic methods (viz. NJ, MP or ML analyses), the branching of *Caulobacterales *with respect to *Rhizobiales *and *Rhodobacterales *was not resolved. In contrast, using the character compatibility approach, the *Caulobacterales *and related species were found to consistently branch in between the *Rhizobiales *and the *Rhodobacterales *(Fig. 2). Previously, this approach has also proven useful in clarifying the phylogenetic placement of *Salinibacter ruber*, which was not resolved by other methods [33,59].

The phylogenetic studies presented here reveal that a number of species belonging to the *Hyphomonadaceae *family (viz. *M. maris, O. alexandrii *and *H. neptunium*), for which sequence information is available and that are presently grouped with the *Rhodobacterales*, branch reliably with the *Caulobacterales *(Figs 1 and 2)[29,47]. The same is also true for *P. bermudensis*, which is the only species from *Parvularculales *order, for which sequence information is available. The grouping of these species with the *Caulobacterales *is independently strongly supported by phylogenomic studies, where a number of proteins that are unique for *C. crescentus *were present in these species and at the same time many proteins that are distinctive characteristics of the *Rhodobacterales *were absent in them. These results make a strong case for the transfer of these *Hyphomonadaceae *species and also *P. bermudensis *to an expanded *Caulobacterales *order [29,47]. Another taxonomic anomaly identified by the present study concerns the phylogenetic position of *Stappia aggregata*. This species was originally identified as an *Agrobacterium*-related species, but later transferred to the *Rhodobacterales *order [43]. However, the shared presence of many *Rhizobiales *-specific proteins by *S. aggregata *and the absence of various *Rhodobacterales*-specific proteins in it, strongly suggest that it should be regrouped with the *Rhizobiaceae-Phyllobacteriacea *species.

The overwhelming majority of the identified α-proteobacteria-specific proteins do not have a homolog showing significant similarity in any other bacteria. The group-specificities of these proteins indicate that their genes have evolved in a common ancestor of these particular groups or clades of α-proteobacteria (Fig. 3). The clade specificities of these proteins also provide evidence that following their evolution, their genes have been transmitted primarily in a vertical manner, and that non-specific mechanisms such as LGTs have not played a significant role in their species distribution. Similar inferences have been reached in earlier studies for proteins that are specific for other higher taxa of bacteria [20,22-24,33,60]. Most of the α-proteobacteria-specific proteins identified in the present work are of unknown function. A number of these proteins are present in the genomes in clusters of two or three, suggesting that they may form functional units and could be involved in related functions [18,26,48,61]. The retention of these α-proteobacteria-specific proteins and conserved indels by the indicated clades of α-proteobacteria over long evolutionary periods strongly suggests that they serve essential functions in these groups of bacteria. Hence, studies on their cellular functions may lead to the discovery of novel biochemical and physiological characteristics that are distinctive characteristics of either all α-proteobacteria or their particular subgroups. Lastly, the primary sequences of many of these genes/proteins are highly conserved and they provide novel means for the identification and characterization of these bacteria by PCR-based and immunological methods.

## Methods

### Identification of proteins that are specific for alpha proteobacteria

The Blastp searches were carried out on each ORF in the genomes of *B. japonicum *USDA 110, *B. suis *1330, *C. crescentus *CB15, *G. oxydans *621H, *M. loti *MAFF303099, *N. winogradskyi *Nb-255, *N. aromaticivorans *DSM 12444, *R. sphaeroides *2.4.1, *Silicibacter sp*. TM1040, *R. rubrum *ATCC 11170 and *Wolbachia *(*D. melanogaster*) endosymbiont to identify proteins that are uniquely present in α-proteobacteria species at different phylogenetic depths. The blast searches were performed against all organisms (i.e. non-redundant (nr) database) using the default parameters, without the low complexity filter [62]. The proteins that were of interest were those where either all significant hits were from the indicated groups (or orders) of α-proteobacteria, or which involved a large increase in E values from the last hit belonging to a particular group to the first hit from any other group and the E values for the latter hits were > 1e ^-4^, indicating weak similarity that could occur by chance. However, higher E values were often considered significant for smaller proteins as the magnitude of the E value depends upon the length of the query sequence [62]. All promising proteins were further analyzed using the position-specific iterated (PSI)-Blast program [62]. In this study, we have also retained a few proteins where 1 or 2 isolated species from other groups had acceptable E values, as they provide possible cases of LGTs. For all of the proteins that are specific for α-proteobacteria, their protein ID's, accession numbers and any information regarding cellular functions (such as COG number or presence of any conserved domain) were tabulated and are presented. In describing various proteins in the text, "bll, bsl, blr", "BQ", "BR or BRA" "CC," "GOX" "ml", "Nwi", "Saro", "RSP", "TM1040", "Rru" and "WD" indicate the identification numbers of the proteins in the genomes of *B. japonicum*, *Bartonella quintana*, *B. suis*, *C. crescentus*, *G. oxydans*, *M. loti*, *N. winogradskyi *Nb-255, *N. aromaticivorans*, *R. sphaeroides *2.4.1, *Silicibacter sp*. TM1040, *R. rubrum *and *Wolbachia *(*Dros*.) endosymbiont, respectively.

### Phylogenetic analysis

The amino acid sequences for 12 conserved proteins (viz. RNA polymerase β and β' subunits, alanyl-tRNA synthetase, phenyalanyl-tRNA synthetase, arginyl-tRNA synthetase, protein synthesis elongation factors EF-Tu and EF-G, RecA, Gyrase A, Gyrase B, Hsp60 and Hsp70) for different species were downloaded from the NCBI database and aligned using the CLUSTAL × program [63]. In addition to the sequences for 50 α-proteobacteria species, sequences for two deep-branching species viz. *Helicobacter pylori *and *Campylobacter jejuni *[58], were also included for rooting purposes. The sequence alignments for all 12 proteins were concatenated into a single large file and poorly aligned regions were removed using the Gblocks 0.91b program [64]. The final sequence alignment that was used for phylogenetic analyses contained 7652 aligned positions. A neighbour-joining tree based on this alignment was constructed based on Kimura's two parameter model distances using the TREECON program [65]. Maximum-likelihood and MP trees were computed using the WAG+F model plus a gamma distribution with four categories using the TREE-PUZZLE [66] and Mega 3.1 program [67], respectively. All trees were bootstrapped 100 times [68], unless otherwise indicated.

The character compatibility analysis was performed on a concatenated sequences for the above 12 proteins for 25 α-proteobacteria species representing all its main orders plus two outgroup species (i.e. *H. pylori *and *C. jejuni*) [32]. Using the program "DUALSITE" [32], all sites in the sequence alignments where only two amino acid states were found, with each state present in at least two species, were selected. All columns where any gap was present in any of the species were omitted. The useful two state sites were converted into a binary file of "0, 1" characters using the DUALSITE program and this file was used for compatibility analysis [32]. The compatibility analysis was carried out using the CLIQUE program from the PHYLIP (ver. 3.5c) program package [68] to identify the largest clique(s) of compatible characters. The cliques were drawn and the numbers of characters that distinguished different nodes were indicated.

### Identification of conserved indels specific for α-proteobacteria subgroups

Multiple sequence alignments for various proteins constructed in this work were visually inspected to search for any indels in a conserved region that was unique to particular subgroups or orders of α-proteobacteria. The group-specificity of any indel was evaluated by carrying out blast searches on a short segment of the sequence (between 80–120 aa) containing the indel and flanking conserved regions against the non-redundant database. The sequence information for various α-proteobacteria was compiled into signature files that are presented. Sequence information for all other groups of bacteria, which lack these inserts, is not shown.

## Authors' contributions

The initial blastp searches on various genomes were carried out by RSG with the computer assistance provided by Venus Wong. AM analyzed the results of these blast searches to identify various group-specific proteins and confirmed their specificities by means of PSI-blast and genomic blasts. Phylogenetic analyses and identification of conserved indels was done by RSG. RSG was also responsible for directing this study, for final evaluation of the results, and for writing this manuscript. All authors have read and approved the submitted manuscript.

## Supplementary Material

Additional file 1 Proteins that are specific for the *Brucella *species. Many of these proteins are specific for all sequenced Brucella species (viz. *B. abortus, B. melitensis, B. ovis *and *B. suis*), whereas others are present in only 2 or 3 of these species. The proteins only found in a single *Brucella *species are not listed here. Click here for file

Additional file 2 *Bradyrhizobiaceae*-specific proteins that are missing in some species. For the proteins listed in this table, all significant hits in Blastp and PSI-Blast searches are from Bradyrhizobiaceae species. However, unlike the proteins listed in Table 3, which are present in all sequenced *Bradyrhizobiaceae *species belonging to the genera *Bradyrhizobium, Nitrobacter *and *Rhodopseudomonas*, these proteins are generally missing in species from one of these three genera. Click here for file

Additional file 3 Proteins that are specific for the *Sphingomonadales *but missing in one or more species. The proteins listed in part (A) of this table are present in three of the following four *Sphingomonadales *species (Novosphingobium, Erythrobacter, Sphingomonas and Sphingopyxis), where as those listed in part (B) are present in *Novosphingobium *and either *Sphingomonas *or *Erythrobacter*. Click here for file
