# Supplementary material for: Phylogenomics and signature proteins for the alpha Proteobacteria and its main groups
Source: BMC Microbiol. 2007 Nov 28;7:106. doi: 10.1186/1471-2180-7-106 (PMC2241609; doi:10.1186/1471-2180-7-106)
Supplement: Additional file 1 — Proteins that are specific for the Brucella species. Many of these proteins are specific for all sequenced Brucella species (viz. B. abortus, B. melitensis, B. ovis and B. suis), whereas others are present in only 2 or 3 of these species. The proteins only found in a single Brucella species are not listed here. [file 1471-2180-7-106-S1.pdf]

Additional file 1: Proteins that are specific for the *Brucella* species

Present in all species [a]	Missing in 1 of the Brucella species[b]			Missing in 2 species[c]
**Protein ID (Accession)**	**Protein ID(Accession)**	**Protein ID (Accession)**	**Protein ID (Accession)**	**Protein ID (Accession)**
BR0584 (AAN29515)	BR0126 (AAN29082)	BR1674 (AAN30574)	BRA1075 (AAN34242)	BR0447 (AAN29390)
BR0718 (AAN29647)	BR0198 (AAN29150)	BR1694 (AAN30594)	BRA1153 (AAN34312)	BR0624 (AAN29553)
BR0761 (AAN29690)	BR0209 (AAN29158)	BR1696 (AAN30596)	BRA1191 (AAN34349)	BR0638 (AAN29567)
BR0798 (AAN29727)	BR0228 (AAN29177)	BR1709 (AAN30609)	BR1998 (AAN30888)	BR0688 (AAN29617)
BR0820 (AAN29749)	BR0293 (AAN29242)	BR1726 (AAN30625	BRA0493 (AAN33685)	BR0795 (AAN29724)
BR0903 (AAN29831)	BR0398 (AAN29344)	BR1751 (AAN30650)	BRA0736 (AAN33918)	BR0834 (AAN29763)
BR0921 (AAN29847)	BR0675 (AAN29604)	BR1765 (AAN30664)	BRA0741 (AAN33923)	BR1146 (AAN30066)
BR1185 (AAN30105)	BR0551 (AAN29482)	BR1768 (AAN30667)	BR0164 (AAN29117)	BR1171 (AAN30091)
BR1203 (AAN30122)	BR0567 (AAN29498)	BR1928 (AAN30820)	BR0208 (AAN29157)	BR1240 (AAN30159)
BR1239 (AAN30158)	BR0632 (AAN29561)	BR1946 (AAN30838)	BR0485 (AAN29428)	BR1323 (AAN30237)
BR1315 (AAN30232)	BR0641 (AAN29570)	BR2000 (AAN30890)	BR0753 (AAN29682)	BR1348 (AAN30262)
BR1280 (AAN30198)	BR0645 (AAN29574)	BR2011 (AAN30901)	BR0913 (AAN29841)	BR1355 (AAN30269)
BR1384 (AAN30297)	BR0650 (AAN29579)	BR2104 (AAN30994)	BR1625 (AAN30530)	BR1470 (AAN30381)
BR1402 (AAN30315)	BR0662 (AAN29591)	BRA003 (AAN33215)	BR1635 (AAN30538)	BR1577 (AAN30483)
BR1405 (AAN30318)	BR0676 (AAN29605)	BRA0004 (AAN33216)	BR1924 (AAN30816)	BR1597 (AAN30502)
BR1457 (AAN30368)	BR0711 (AAN29640)	BRA0053 (AAN33265)	BR1952 (AAN30844)	BR1624 (AAN30529)
BR1519 (AAN30429)	BR0742 (AAN29671)	BRA0102 (AAN33312)	BRA0179 (AAN33386)	BR1665 (AAN30566)
BR1565 (AAN30474)	BR0764 (AAN29693)	BRA0140 (AAN33348)	BRA0617 (AAN33806)	BR1735 (AAN30634)
BR1592 (AAN30497)	BR0800 (AAN29729)	BRA0150 (AAN33357)	BRA0796 (AAN33975)	BR1858 (AAN30753)
BR1644 (AAN30546)	BR0821 (AAN29750)	BRA0200(AAN33406)	BRA0835 (AAN34010)	BR1867 (AAN30762)
BR1664 (AAN30565)	BR0846 (AAN29775)	BRA0273 (AAN33474)	BRA0837 (AAN34012)	BR1999 (AAN30889)
BR1682 (AAN30582)	BR0941 (AAN29867)	BRA0319 (AAN33519)	BR0719 (AAN29648)	BR2023 (AAN30913)
BR1712 (AAN30612)	BR0947 (AAN29873)	BRA0320 (AAN33520)	BRA0132 (AAN33340)	BR2038 (AAN30928)
BR1797 (AAN30692)	BR0963 (AAN29888)	BRA0330 (AAN33530)	BR0644 (AAN29573)	BR2134 (AAN31024)
BR1823 (AAN30718)	BR0965 (AAN29890)	BRA0337 (AAN33537)	BR1990 (AAN30880)	BR2144 (AAN31034)
BR1942 (AAN30834)	BR0968 (AAN29893)	BRA0342 (AAN33540)	BRA0484 (AAN33677)	BRA0002 (AAN33214)
BR1949 (AAN30841)	BR0973 (AAN29898)	BRA0387 (AAN33585)	BRA0611 (AAN33800)	BRA0197 (AAN33403)
BR1964 (AAN30855)	BR0985 (AAN29908)	BRA0452 (AAN33646)		BRA0198 (AAN33403)
*BR2182 (AAN31071)	BR0987 (AAN29910)	BRA0482 (AAN33674)		BRA0230 (AAN33432)
BRA0176 (AAN33383)	BR0988 (AAN29911)	BRA0493 (AAN33685)		BRA0242 (AAN33444)
BRA0259 (AAN33461)	BR0994 (AAN29917)	BRA0515 (AAN33707)		BRA0287 (AAN33487)
BRA0312 (AAN33512)	BR1009 (AAN29931)	BRA0529 (AAN33721)		BRA0303 (AAN33503)
BRA0313 (AAN33513)	BR1023 (AAN29945)	BRA0539 (AAN33731)		BRA0495 (AAN33687)
BRA0324 (AAN33524)	BR1037 (AAN29958)	BRA0541 (AAN33733)		BRA0498 (AAN33690)
BRA0441 (AAN33635)	BR1050 (AAN29970)	BRA0593 (AAN33782)		BRA0619 (AAN33808)
BRA0592 (AAN33781)	BR1065 (AAN29985)	BRA0629 (AAN33818)		BRA0620 (AAN33809)
BRA0603 (AAN33792)	BR1096 (AAN30016)	BRA0662 (AAN33851)		BRA0705 (AAN33890)
BRA0623 (AAN33812)	BR1135 (AAN30055)	BRA0664 (AAN33852)		BRA0747 (AAN33929)
BRA0668 (AAN33855)	BR1155 (AAN30075)	BRA0674 (AAN33861)		BRA0821 (AAN33996)
BRA0827 (AAN34002)	BR1501 (AAN30412)	BRA0694 (AAN33880)		BRA0950 (AAN34121)
BRA0830 (AAN34005)	BR1253 (AAN30172)	BRA0736 (AAN33918)		BRA1030 (AAN34198)
BRA0959 (AAN34130)	BR1257 (AAN30175)	BRA0741 (AAN33923)		BRA1040 (AAN34207)
BRA0169 (AAN33376)	BR1267 (AAN30185)	BRA0767 (AAN33949)		BRA0133 (AAN33341)
	BR1331 (AAN30245)	BRA0832 (AAN34007)		BRA0164 (AAN33371)
	BR1341 (AAN30255)	BRA0848 (AAN34022)		BR0541 (AAN29472)
	BR1382 (AAN30295)	BRA0856 (AAN34029)		BR0999 (AAN29922)
	BR1409 (AAN30322)	BRA0863 (AAN34036)		BR1101 (AAN30021)
	BR1423 (AAN30336)	BRA0877 (AAN34050)		BR1184 (AAN30104)
	BR1548 (AAN30455)	BRA0895 (AAN34067)		BR1252 (AAN30171)
	BR1567 (AAN30473)	BRA0917 (AAN34089)		BR1352 (AAN30266)
	BR1603 (AAN30508)	BRA0946 (AAN34117)		BR1393 (AAN30306)
	BR1607 (AAN30512)	BRA0977 (AAN34148)		BR1783 (AAN30680)
	BR1633 (AAN30536)	BRA0980 (AAN34150)		BRA0380 (AAN33578)
	BR1645 (AAN30547)	BRA1016 (AAN34184)		BRA0698 (AAN33883)
	BR1663 (AAN30564)	BRA1071 (AAN34238)		BR1569 (AAN30475)

[a] Present in all available *Brucella* species (*B. abortus*,*B. melitensis*, *B. ovis* and *B. suis*)
[b] Present in three of the four available *Brucella* species
[c] Present in two of the four available Brucella species
*BR2182(AAN31071): pilus biosynthesis protein-related protein
