# Supplementary material for: Phylogenomics and signature proteins for the alpha Proteobacteria and its main groups
Source: BMC Microbiol. 2007 Nov 28;7:106. doi: 10.1186/1471-2180-7-106 (PMC2241609; doi:10.1186/1471-2180-7-106)
Supplement: Additional file 2 — Bradyrhizobiaceae-specific proteins that are missing in some species. For the proteins listed in this table, all significant hits in Blastp and PSI-Blast searches are from Bradyrhizobiaceae species. However, unlike the proteins listed in Table 3, which are present in all sequenced Bradyrhizobiaceae species belonging to the genera Bradyrhizobium, Nitrobacter and Rhodopseudomonas, these proteins are generally missing in species from one of these three genera. [file 1471-2180-7-106-S2.pdf]

Additional file 2: Bradyrhizobiaceae-specific proteins that are missing in some species

**Proteins found in only two of the three genera (*Bradyrhizobium, Nitrobacter* and R*hodopseudomonas)***					
Gene ID	Accession Number	Function	Gene ID	Accession Number	Function
bll1585 [1,2]	NP_768225	Phage integrase	blr0712 [3]	NP_767352	Hypothetical
bll2067 [1,2]	NP_768707	NfeC	blr0806 [3]	NP_767446	Hypothetical
bll5079 [1,2]	NP_771719	Hypothetical	blr1300	NP_767940	Hypothetical
blr3860 [1]	NP_770500	Hypothetical	blr1325	NP_767965	Hypothetical
bsl6289 [1,2]	NP_772929	Hypothetical	blr3983 [2, 3]	NP_770623	Hypothetical
bsr7049 [1]	NP_773689	Hypothetical	blr5578	NP_772218	Hypothetical
bsr7348 [1]	NP_773988	Hypothetical	blr5744 [2,3]	NP_772384	Hypothetical
Nwi_1474	YP_318087	Per-hexamer repeat gene protein	blr5768 [3]	NP_772408	Hypothetical
Nwi_1489 [1,2]	YP_318102	Hypothetical	blr7338 [2,3]	NP_773978	Hypothetical
Nwi_1537	YP_318150	Hypothetical	blr7339 [2,3]	NP_773979	Hypothetical
Nwi_1618 [1,2]	YP_318231	Hypothetical	blr7579 [2,3]	NP_774219	Hypothetical
Nwi_1639	YP_318252	Hypothetical	blr7780 [3]	NP_774420	Hypothetical
Nwi_2369 [1]	YP_318975	Hypothetical	bsl4913	NP_771553	Hypothetical
Nwi_2372 [1,4]	YP_318978	Hypothetical	bsl5034	NP_771674	Hypothetical
Nwi_2373[1,4]	YP_318979	Hypothetical	bsr0093 [3]	NP_766733	Hypothetical
Nwi_2462	YP_319067	Hypothetical	bsr0398	NP_767038	Hypothetical
Nwi_2768	YP_319373	Hypothetical	bsr4225	NP_770865	Hypothetical
Nwi_2909 [1,2]	YP_319511	Hypothetical	Nwi_0650 [3]	YP_317268	Hypothetical
Nwi_3011[4]	YP_319613	Hypothetical	Nwi_0709 [3]	YP_317327	Hypothetical
bll0660 [3]	NP_767300	Hypothetical	Nwi_1118 [3]	YP_317732	Hypothetical
bll5324 [3]	NP_771964	Hypothetical	Nwi_1164 [1,3]	YP_317778	Hypothetical
bll5535 [3]	NP_772175	Hypothetical	Nwi_1168	YP_317782	Hypothetical
bll5766 [3]	NP_772406	Hypothetical	Nwi_1301 [3]	YP_317914	Hypothetical
bll7047	NP_773687	Hypothetical	Nwi_2399 [1]	YP_319005	Hypothetical
bll8113	NP_774753	Hypothetical	Nwi_2415 [3]	YP_319021	Hypothetical
bll8136	NP_774776	Hypothetical	Nwi_3107 [3]	YP_319706	Hypothetical
blr0497	NP_767137	Hypothetical			

[1] Missing in one or more species of *Nitrobacter*
[2] Missing in *Bradyrhizobium* sp. BTAi1
[3] Missing in one or more strains of *Rhodopseudomonas*
[4] Missing in Bradyrhizobium japonicum USDA 110
