# Supplementary material for: Phylogenomics and signature proteins for the alpha Proteobacteria and its main groups
Source: BMC Microbiol. 2007 Nov 28;7:106. doi: 10.1186/1471-2180-7-106 (PMC2241609; doi:10.1186/1471-2180-7-106)
Supplement: Additional file 3 — Proteins that are specific for the Sphingomonadales but missing in one or more species. The proteins listed in part (A) of this table are present in three of the following four Sphingomonadales species (Novosphingobium, Erythrobacter, Sphingomonas and Sphingopyxis), where as those listed in part (B) are present in Novosphingobium and either Sphingomonas or Erythrobacter. [file 1471-2180-7-106-S3.pdf]

Additional file 3: Proteins that specific for the *Sphingomonadales* but missing in one or more species

A. Proteins Uniquely Present in Three of the Four Sphingomonadales Genera (Novosphingobium, Erythrobacter, Sphingomonas or Sphingopyxis)

Saro_0166	YP_495449	Hypothetical	Saro_2373	YP_497643	Hypothetical
Saro_0206	YP_495488	Hypothetical	Saro_2384[3]	YP_497654	Hypothetical
Saro_0269	YP_495551	Hypothetical	Saro_2385[3]	YP_497655	Hypothetical
Saro_0968	YP_496247	Hypothetical	Saro_2523	YP_497793	Hypothetical
Saro_0881	YP_496160	Hypothetical	Saro_2643	YP_497913	Hypothetical
Saro_1030	YP_496309	Hypothetical	Saro_2670	YP_497940	Hypothetical
Saro_1159	YP_496438	Hypothetical	Saro_3064	YP_498333	Hypothetical
Saro_1239	YP_496518	Hypothetical	Saro_3117	YP_498386	Hypothetical
Saro_1363	YP_496641	Hypothetical	Saro_3124	YP_498393	Hypothetical
Saro_1502	YP_496777	Hypothetical	Saro_3131	YP_498400	Hypothetical
Saro_1594	YP_496868	Hypothetical	Saro_3144	YP_498413	Hypothetical
Saro_1978	YP_497252	Hypothetical	Saro_3302	YP_498571	Hypothetical
Saro_2194	YP_497467	Hypothetical			

B. Proteins Unique Present in *Novosphingobium* and either *Sphingomonas* or *Erythrobacter*

Gene ID	Accession Number	Function	Gene ID	Accession Number	Function
Saro_0916	YP_496195	Hypothetical	Saro_2126	YP_497399	Hypothetical
Saro_1581	YP_496855	Hypothetical	Saro_2154	YP_497427	Hypothetical
Saro_1633	YP_496907	Hypothetical	Saro_2185	YP_497458	Hypothetical
Saro_2089	YP_497362	Hypothetical	Saro_2188	YP_497461	Hypothetical
Saro_3076	YP_498345	Hypothetical	Saro_2270	YP_497541	Hypothetical
Saro_3091	YP_498360	Hypothetical	Saro_2271	YP_497542	Hypothetical
Saro_3311	YP_498580	Hypothetical	Saro_2562	YP_497832	Hypothetical
Saro_0027	YP_495310	Hypothetical	Saro_2563	YP_497833	Hypothetical
Saro_0243	YP_495525	Hypothetical	Saro_2781	YP_498051	Hypothetical
Saro_0592	YP_495873	Hypothetical	Saro_2856	YP_498126	Hypothetical
Saro_1004	YP_496283	Hypothetical	Saro_3056	YP_498325	Hypothetical
Saro_1294	YP_496572	Hypothetical	Saro_3159	YP_498428	Hypothetical
Saro_1767	YP_497041	Hypothetical	Saro_3222	YP_498491	Hypothetical
Saro_1911	YP_497185	TadE-like	Saro_3248	YP_498517	Hypothetical
Saro_1959	YP_497233	Hypothetical	Saro_3323	YP_498592	Hypothetical
Saro_2035	YP_497308	Hypothetical	Saro_3326[1]	YP_498595	Hypothetical

[1] Caulobacter crescentus CB15 is found to be significant
